# Safety of wide-awake local anesthesia with no tourniquet (WALANT) in for lower limb surgery: A potential alternative in times of emergency

**DOI:** 10.3389/fsurg.2022.848422

**Published:** 2022-09-09

**Authors:** Mohd Yazid Bajuri, Nur Sai’dah Saidfudin, Norliyana Mazli, Nik Alif Azriq, Aina Fatini Azemi

**Affiliations:** Department of Orthopaedics and Traumatology, Faculty of Medicine, National University of Malaysia, Kuala Lumpur, Malaysia

**Keywords:** WALANT, local anaesthesia, ambulatory surgery, pain, satisfaction

## Abstract

Lower limb surgery is usually performed under general or regional anaesthesia in normal operating room setting. However, when the surgery need to be performed in situations where there are limited resources and equipment, especially during a pandemic outbreak, in battlefields or area of disaster occurrence, the wide-awake local anaesthesia no tourniquet (WALANT) surgery can be utilised. This study aims to assess the efficacy of performing lower limb surgery using WALANT technique. A randomised cross-sectional study was designed to assess the effectiveness of WALANT in lower limb surgery, particularly in terms of duration of anaesthesia, Hamilton Anxiety Rating Scale (HAM-A), pain visual analogue scale (VAS), duration of surgery, amount of estimated blood loss (EBL) and total length of stay (LOS). A total of 91 patients requiring lower limb surgery were recruited, with only 83 patients completed the VAS pain assessment for all time points of the study. Mean age of patients was 52.1 ± 14.9 years. Mean VAS score were 1.19 ± 1.53 and 1.46 ± 1.86, preoperatively and intraoperatively. Mean VAS score were 0.55 ± 1.52, 0.60 ± 1.41, and 1.06 ± 1.69 at 2, 4, and 6 h post-surgery, respectively. Majority (79; 86.8%) of patient has preoperative anxiety score that was <17. Mean surgery duration was 65.28 ± 39.02 min, mean EBL was 91.34 ± 78.94 cc, whereas mean LOS was 3.35 ± 1.16 days. EBL was a weak predictor of postoperative pain. In conclusion, WALANT technique for lower limb surgery is effective and safe.

## Introduction

Lower limb surgery is commonly done in general orthopaedic setting following various trauma or morbidities such as motor vehicle accident, assaults, fall, foot deformity and others. It is usually performed under general or regional anaesthesia. Apart from the inherent risks and costs associated with these anaesthetic techniques (1), it is usually difficult to perform these types of surgeries when there are limited resources and equipment available, especially during a pandemic outbreak such as COVID-19 where there is a shift of hospital resources to focus on pandemic management. Therefore, an alternative approach should be explored to improve these limitations. One of the surgical approaches which could improve the healthcare service is the wide-awake local anaesthesia no tourniquet (WALANT) surgery. WALANT refers to a type of surgery which involves surgeon-administered mixture of local anaesthetic for pain control and epinephrine for hemostasis and improved anaesthetic effect. Since no tourniquet, sedation or general anaesthesia are used for the surgery, the patient remains fully awake throughout the entire surgical procedure (2).

WALANT has been used in hand surgeries (3, 4) as well as procedures involving foot and ankle (1, 2). This anaesthetic technique has fewer risks and side effects compared to conventional sedation or general anaesthesia technique. For example, preoperative testing is often unnecessary and no anaesthetic staff or equipment are required. Since there is limited equipment needed, the technique can be used for ambulatory clinic where surgeries can be performed in any places. This is beneficial in certain settings such as in the battlefield and areas where disaster occurs. Besides, surgeon can communicate with the sober and coherent patient throughout perioperative period, providing the patient with greater insight into both the procedure and the recovery process (1).

Despite the benefits, the patient’s comfort and well-being have been the main concern for surgeons given the nature of WALANT surgery. As such, several studies had been done to assess patient experience with WALANT technique and the results were rather encouraging. For example, in a 2014 trial for WALANT in the lower limb surgery setting which involved the forefoot, hind foot and lower leg, the patients were assessed for their level of anxiety, pain and satisfaction intraoperatively. Results from the trial showed that peri- and post-operative pain and anxiety were significantly improved in patients who underwent WALANT surgery compared to the general anaesthesia group (1). Another different survey reported that the patients would choose WALANT technique if another operation was necessary and they would recommend the technique to a friend (5).

Although WALANT surgery is widely used in other countries, it is still uncommon in Malaysia. Since there is no available data published for WALANT lower limb surgery in the country, this study aims to assess the efficacy of performing lower limb surgeries (from knee region and distally) using the WALANT technique. The efficacy of the technique is assessed by studying the effectiveness of WALANT anaesthesia over the surgery, duration of action of the anaesthesia, duration of surgery performed, amount of estimated blood loss (EBL) during the surgery as well as the total length of stay (LOS) at the hospital after the surgery for the patients. This study would be the pioneer project in Malaysia and would contribute further in the emerging WALANT technique, especially in lower limb surgery.

## Methods

### Study design

This is a randomised cross-sectional study performed during the period of July 2020 to June 2021 in the General Operation Theatre (GOT), Emergency Department Operation Theatre (EDOT) of Daycare of Hospital Canselor Tuanku Mukhriz (HCTM), Pusat Perubatan Universiti Kebangsaan Malaysia (PPUKM). The study conformed to the Declaration of Helsinki and followed International Conference on Harmonization (ICH) – Good Clinical Practice (GCP) guidelines. Assessment of efficacy on WALANT surgery would be based on all subjects who completed the study.

### Patient selection

Patients with lower limb surgeries, from knee and distally, who agreed to use the WALANT method, were included in the study. Patients with peripheral vascular disease (ankle brachial systolic index, ABSI <0.9 and >1.2), poorly controlled diabetes mellitus, ischaemic heart disease, polytrauma, injuries involving femur or above knee level were excluded from the study. Patients with lower limb infections or who had surgeries involving intramedullary devise such as nailing were also excluded. Besides, psychiatry patients like those with anxiety were also excluded from the study. Informed consent was obtained from all the patients before participating in the study.

### Preparation and administration of WALANT solution

The WALANT solution consisted of 50 ml of lignocaine (1%) with 1:100,000 epinephrine. It was prepared by using 50 ml of normal saline (0.9%) and 50 ml of lignocaine (2%). 1:100,000 epinephrine was prepared by mixing 100 ml of the prepared solution with 1 ml of 1:1,000 epinephrine.

The toxicity of lignocaine is 7 mg/kg (6). Therefore, the maximum amount of WALANT solution to be administered in a patient had to be calculated based on the patient’s weight. For example, a patient weighs 70 kg can tolerate 490 mg of lignocaine (1%) (70 kg × 7 mg/kg). Since 1 ml of WALANT solution consisted of 10 mg of lignocaine (1%) with 1:100,000 epinephrine, the maximum amount of WALANT solution to be administered in the patient is 49 ml.

10 ml of sodium bicarbonate (8.4%) was added along with the WALANT solution to reduce pain during administration before the lower limb surgery begun. The solution was injected subdermally in perpendicular direction into area of surgery.

Other drugs such as phentolamine, terbutaline or phenoxybenzamine, which can reverse epinephrine vasoconstriction in human digits; and intraipid 20% solution, which can reverse lignocaine toxicity, should be prepared in the emergency trolley in case of any complication occur.

### Data collection and analysis

**Figure 1:**
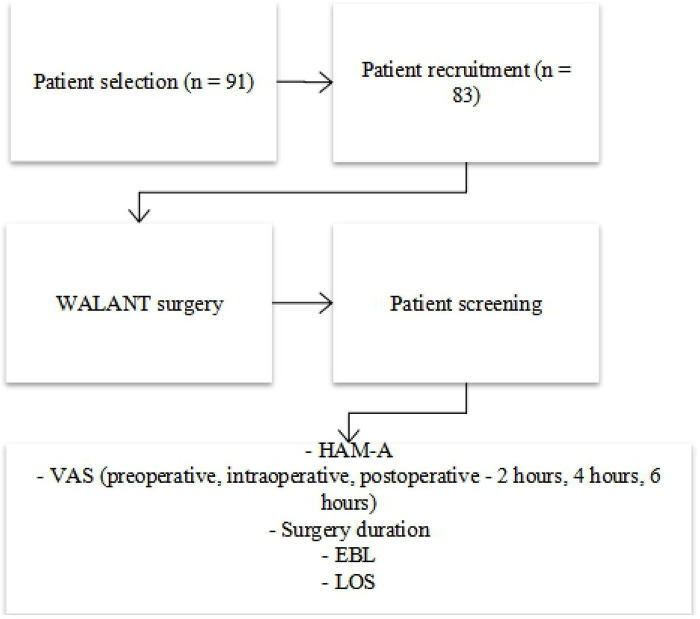
Study flow chart.

Various data including patients’ demographics, Hamilton Anxiety Rating Scale (HAM-A), pain visual analogue scale (VAS), duration of surgery in minutes, EBL during the surgery in cubic centimeters (cc) and total LOS in hospital (days) were recorded. The data were analysed using the Rasch Measurement Analysis Software version 4.4.5. Level of significance was set at *p* < 0.05 for every analysis. Figure 1 shows the flow chart of the study.

## Results

### Patient demographics

**Figure 2:**
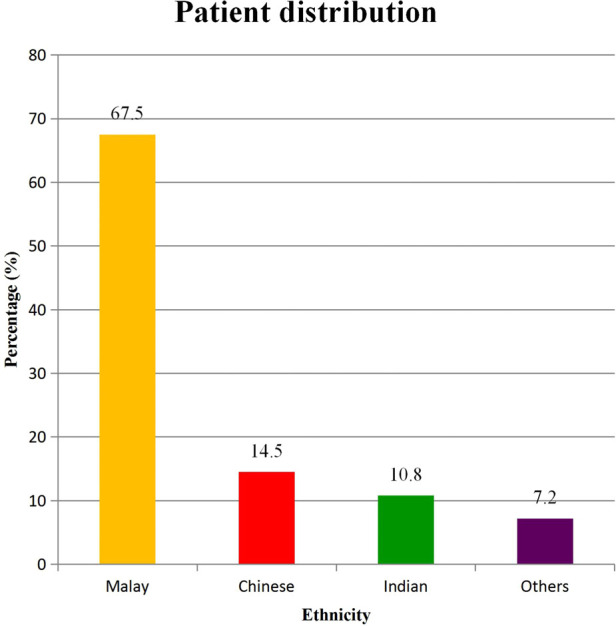
Percentage of patient (%) according to ethnicity.

A total of 91 (34 males and 57 females) patients requiring lower limb surgery were recruited in this study. Only 83 (32 males and 51 females) patients completed the VAS pain assessment for all time points of the study with a mean age of 52.1 ± 14.9 years (Figure 1). Race distribution for these patients were 56 (67.5%) Malay, 12 (14.5%) Chinese, 9 (10.8%) Indian, and 6 (7.2%) other races (Figure 2).

**Figure 3:**
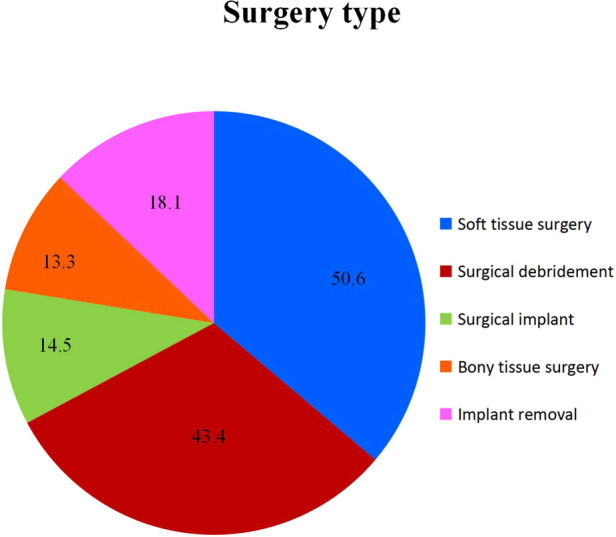
Percentage of patient (%) according to surgery type.

**Figure 4:**
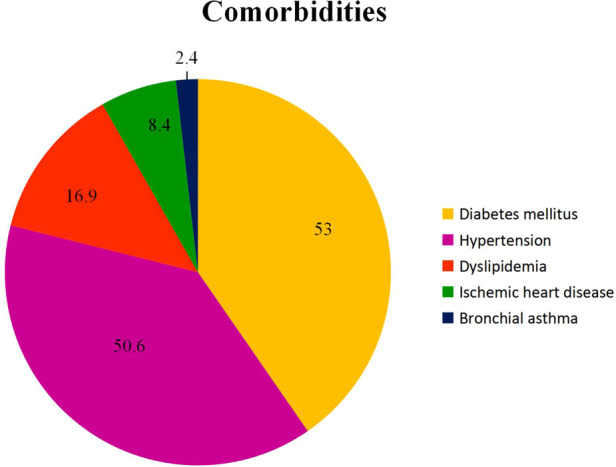
Percentage of patient (%) according to comorbidity.

Out of the total 83 patients, 14.5% (12) patients required surgical implant, 43.4% (36) patients required surgical debridement of the wound, 50.6% (42) patients required soft tissue surgery, 13.3% (11) patients required bony tissue procedure and 18.1% (15) patients required removal of implant (Figure 3). In terms of comorbidities, 50.6% (42) patients had hypertension, 53.0% (44) patients had diabetes mellitus, 16.9% (14) patients had dyslipidemia, 8.4% (7) had ischemic heart disease, and 2.4% (2) had bronchial asthma (Figure 4). Additionally, the mean fasting blood sugar (FBS) and ABSI among the patients were known to be 7.5 ± 2.2 and 1.1 ± 0.2, respectively.

### Pain assessment Pre-, intra- and post-surgery

Pain level was assessed before, during, and after WALANT surgery with VAS. The mean VAS score pre-surgery was 1.19 ± 1.53. Reasonably, pain was greater during surgery but remains at low level with the intra-surgery VAS score of 1.46 ± 1.86. The observed low level of pain was maintained after surgery with VAS score of 0.55 ± 1.52, 0.60 ± 1.41, and 1.06 ± 1.69 at 2, 4, and 6 h post-surgery, respectively. No statistically significant difference between the time points were observed.

### Pre-surgical anxiety scale, duration of surgery, blood loss and length of stay at hospital

Other surgical outcome parameters measured to evaluate the effectiveness of WALANT surgery include preoperative anxiety, duration of surgery, blood loss, and LOS at hospital. Majority of 91.6% (76) of the patients scored less than 17 on the HAM-A scale depicting an overall low anxiety before the WALANT surgery. The mean time for the WALANT surgery from the incision to the wound closure was 65.28 ± 39.02 min. The mean estimated blood loss following WALANT surgery was 91.34 ± 78.94 cc. Finally, the mean LOS following WALANT surgery was 3.35 ± 1.16 days.

### Predictors of post-surgical pain

Linear regression analysis was performed to determine the independent predictors associated with pain after 6 h of WALANT surgery. Table 1 describes the association of each risk factor to the surgical pain outcome. Estimated blood loss was found to be correlated with the surgical pain outcome (*p* < 0.05). However, the multivariate regression analysis revealed that blood loss is not a strong predictor of surgical pain.

**Table 1:** Correlation between risk factors and post-surgical pain.

Risk factors	*p*-value
Age	0.806
Gender	0.624
Hypertension	0.473
Diabetes mellitus	0.791
Dyslipidemia	0.267
Ischemic heart disease	0.234
Bronchial asthma	0.566
Fasting blood sugar (FBS)	0.082
Ankle brachial systolic index (ABSI)	0.144
Surgery duration	0.217
Estimated blood loss (EBL)	0.011*
Length of hospital stays (LOS)	0.444

*p < 0.05.

## Discussion

Wide local anasthesia no torniquet technique has been extensively incorporated in upper limb surgery with a number of studies being published especially in the last decade. Compared to that, the usage of WALANT in lower limb operation requires more studies particularly in Malaysia. The surge of COVID-19 infection seems to be timely for the evaluation of WALANT effectiveness in lower limb surgeries due to the shift of resources towards pandemic management.

In various hand surgeries that applied WALANT technique (6–8), it was found that WALANT provides numerous practical benefits as compared to conventional anaesthesia techniques. It is with confidence stemming from the success of these surgeries that this study is conducted to look for equivalent advantages in lower limb operations.

In this study, the results showed a higher intra-surgery pain VAS score of 1.46 ± 1.86 than that of pre-surgery (1.19 ± 1.53). These scores, however, contradict with a previous WALANT study on foot and ankle surgery by MacNeill and Mayich, which reported a lower intra-surgery pain score of 0.75 ± 0.85 compared to preoperative score of 5.00 ± 3.11 (1). But, the pain scores remained stable and low before, during and even several hours after the surgery, as opposed to those recorded in MacNeill and Mayich, which was considerably higher before (5.00 ± 3.11) and after (5.21 ± 2.90) the surgery (1). The stable scores were in line with another WALANT study involving forefoot surgery by Wright et al., which also reported fairly stable scores ranging from 1.65 ± 1.99 (preoperative), 0.17 ± 0.32 (intraoperative) to 0.45 ± 1.10 (postoperative) (2).

Several studies have shown low anxiety in patients undergoing different types of lower limb surgeries. For example, Bilgetekin et al. reported a median VAS anxiety (VAS-A) score of 1 for the use of WALANT foot and ankle surgery resulting from medial malleolus fracture, lateral malleolus fracture, Achilles tendon rupture, proximal phalangeal fracture, Lisfranc injury, syndesmotic injury, deltoid ligament injury and fifth metatarsal fracture (7). Another study reported significantly lower anxiety for patients who received WALANT forefoot surgery (3.20 ± 3.02) compared to patients who received general anaesthesia treatment (2.98 ± 3.21). For the group that received WALANT treatment, the anxiety score decreased considerably from pre-surgery period (3.20 ± 3.02) to post-surgery period (0.08 ± 0.34) (2). Consistent with previous studies, results from this study also reported an overall low anxiety (<17 on HAM-A scale) in the patients before WALANT surgery.

An open reduction and internal fixation surgery of ankle fractures using WALANT technique reported a mean operation time of 59.72 ± 7.19 min (8), which is approximately the same as the duration recorded in this current study (65.28 ± 39.02 min). Another similar study involving ankle operation reported a shorter mean surgery duration of 36.6 ± 7 min (7). The shorter operation time may be due to the simplicity of the ankle surgical procedures performed as opposed to the more complicated procedures performed in this study, including soft tissue surgery, surgical debridement of wound, surgical implantation and removal of implant.

The mean EBL recorded in the current study was 91.34 ± 78.94 cc, which is higher compared to the average amount of blood loss recorded (29.40 ± 7.38 ml) in an ankle fracture fixation surgery using the same WALANT technique (8). This is probably due to the majority of patients (50.6%) in this study underwent soft tissue surgery, which may lead to more blood loss compared to procedures involving ankle fracture fixation. Although the mean amount of blood loss recorded in this study is rather high, it is significantly lower when compared to other lower limb surgery using conventional anaesthesia techniques. For example, transtibial amputation using tourniquet was reported to have caused a median intraoperative blood loss of 200 ml (interquartile range = 100–300 ml) and a median total blood loss of 737 ml (interquartile range = 331–1218) from Day 0 to Day 4 after the surgery (9). Even a less complicated surgery like total knee replacement would cause a mean total blood loss of about 1.0–1.5 liter when general anaesthesia with tourniquet is used (10).

The mean LOS following WALANT surgery recorded in this study was 3.35 ± 1.16 days. This value is longer compared to other studies using the similar WALANT technique. For instance, a study indicated that patients who underwent ankle fracture surgery only spent an average LOS of 8.3 ± 6.1 h at the hospital on the day of surgery before they were discharged (7). Another similar study involving forefoot surgery reported a shorter LOS (3.60 ± 1.23 h) at the hospital (2). This, again, may be due to the simplicity of the surgeries performed in the two studies, unlike those complicated ones performed in the current study that may require longer time for the wounds to heal. Nonetheless, the mean LOS for the current study is considerably shorter in comparison with lower extremity amputation using conventional anaesthesia, which may lead to months of hospitalization because of serious postoperative complications.

Meanwhile, the association of independent predictors with postoperative pain was determined through linear regression analysis. Results published by multiple studies indicated that age, gender, comorbidities such as diabetes mellitus (11, 12) and duration of surgery (13) are risk factors for higher incidence of post-surgical pain. However, results from this study showed that age, gender, surgery duration and comorbidities including hypertension, diabetes mellitus, dyslipidemia, ischemic heart disease and bronchial asthma did not correlate with post-surgical pain. Blood sugar level (14) and ABSI (15) were found to be associated with several postoperative complications such as poor wound healing and post-surgical cardiovascular events. But, linear regression analysis did not show any relation between the two variables and postoperative pain. Next, longer hospital stay is often associated with a higher incidence of postoperative complications including pain and delayed wound healing, especially in lower extremity amputation. However, in this study, the *p*-value from linear regression analysis showed no correlation between LOS and post-surgical pain. Although EBL was the only variable found to be correlated with post-surgical pain, it was not a strong predictor as indicated through multivariate regression analysis. Therefore, it cannot be used as a reliable predictor of postoperative pain in patients undergoing lower limb surgery.

The experiment yields an overall satisfying result in regards to the safety and effectiveness of WALANT technique application in lower limb surgery, as demonstrated by previous studies (16–18). The low and stable pain VAS score, especially hours after the surgery, proves the effectiveness of WALANT anaesthesia solution in pain management during and after the lower limb surgery. Besides, the overall low anxiety score before WALANT surgery also shows that the patients are generally quite tolerant of this type of wide-awake surgery. This enables WALANT surgery to be practised more widely in different lower limb surgical procedures.

Furthermore, the short duration of surgery, low amount of blood loss and LOS in hospital enable WALANT surgery to be carried out in places where there are limited healthcare resources and equipment. Short duration of surgery is an important criterion for the application of WALANT technique in battlefields or area of disaster occurence, where certain surgery needed to perform over a short period of time with limited resources in order to save lives. Next, low amount of blood loss during operation means no or limited blood transfusion is required. This is favourable when surgeries are performed outside of operating rooms without appropriate equipment or resources. Finally, short LOS poses another added advantage for WALANT surgery, especially during times of limited intensive care unit or operating room resources when there is a pandemic like COVID-19. More beds and ventilators can therefore be allocated to patients who are truly in need.

Nonetheless, this study has several limitations. One of which is small sample size. Only 39 patients completed the VAS pain assessment for all time points despite a total of 81 patients recruited in this study. According to the sample size and item calibration or person measure stability of Rasch Model, a minimum of 50 subjects are needed to achieve a 99% confidence interval (CI) in an experiment (19). Hence, it is recommended for the experiment to be conducted on a larger sample size so that a 99% of CI can be achieved on each parameter, thus validating the data. Perhaps, with the increase of sample size and data, predictors of post-surgical pain can be determined. The next limitation is lack of pain and anxiety assessment. Pain assessment should be done during anaesthesia injection and over the period of days after the surgery to allow investigation of the efficacy of WALANT technique over a longer period of time. Anxiety assessment should also be conducted intra- and post-operatively to better monitor the emotional changes in patients.

## Conclusion

WALANT technique for lower limb surgery is as effective as for upper limb surgery, as indicated by low and stable pain VAS score, low preoperative anxiety score on HAM-A, short surgery duration, low amount of estimated blood loss and short LOS at hospital. Age, gender, comorbidities (hypertension, diabetes mellitus, dyslipidemia, ischemic heart disease and bronchial asthma), FBS, ABSI, surgery duration and LOS are not predictors of post-surgical pain while EBL is a weak predictor. Further studies with larger sample size and more parameters included should be conducted to evaluate the safety and efficacy of WALANT technique application in lower limb surgery.

## Data Availability

The original contributions presented in the study are included in the article/Supplementary Material, further inquiries can be directed to the corresponding author/s.

## References

[1] MacNeill AL Mayich DJ. Wide-awake foot and ankle surgery: a retrospective analysis. Foot Ankle Surg. (2017) 23:307–10. 10.1016/j.fas.2016.09.004 29202993

[2] Wright J MacNeill AL Mayich DJ. A prospective comparison of wide-awake local anesthesia and general anesthesia for forefoot surgery. Foot Ankle Surg. (2017) 25:211–4. 10.1016/j.fas.2017.10.015 29409279

[3] Lalonde DH Martin AL. Wide-awake flexor tendon repair and early tendon mobilization in zones 1 and 2. Hand Clin. (2013) 29:207–13. 10.1016/j.hcl.2013.02.009 23660056

[4] Higgins A Lalonde DH Bell M McKee D Lalonde JF. Avoiding flexor tendon repair rupture with intraoperative total active movement examination. Plast Reconstr Surg. (2010) 126:941–5. 10.1097/PRS.0b013e3181e60489 20463621

[5] Teo I Lam W Muthayya P Steele K Alexander S Miller G. Patients’ perspective of wide-awake hand surgery - 100 consecutive cases. J Hand Surg Eur. (2013) 38:992–9. 10.1177/1753193412475241 23348603

[6] Feldman G Orbach H Rinat B Rozen N Rubin G. Internal fixation of metacarpal fractures using wide awake local anesthesia and no tourniquet. Hand Surg Rehabil. (2020) 39(3):214–7. 10.1016/j.hansur.2020.01.003 32070790

[7] Orbach H Rozen N Rubin G. Open reduction and internal fixation of intra-articular distal radius fractures under wide-awake local anesthesia with no tourniquet. J Int Med Res. (2018) 46(10):4269–76. 10.1177/0300060518793036 30111223 PMC6166335

[8] Fulchignoni C Bonetti MA Rovere G Ziranu A Maccauro G Pataia E. Wide awake surgery for flexor tendon primary repair: a literature review. Orthop Rev. (2020) 12(Suppl 1):61–3. 10.4081/or.2020.8668 PMC7459365 32913601

[9] Lalonde D Eaton C Amadio P Jupiter J. Wide-awake hand and wrist surgery: a new horizon in outpatient surgery. Instr Course Lect. (2015) 64:249–59. 25745911

[10] Bilgetekin YG Kuzucu Y Öztürk A Yüksel S Atilla HA Ersan Ö. The use of the wide-awake local anesthesia no tourniquet technique in foot and ankle injuries. Foot Ankle Surg. (2010) 27:535–8. 10.1016/j.fas.2020.07.002 32682691

[11] Wied C Tengberg PT Holm G Kallemose T Foss NB Troelsen A Tourniquets do not increase the total blood loss or reamputation risk in transtibial amputations. World J Orthop. (2017) 8:62–7. 10.5312/wjo.v8.i1.62 28144581 PMC5241547

[12] Magill P Cunningham EL Hill JC Beverland DE. Identifying the period of greatest blood loss after lower limb arthroplasty. Arthroplast Today. (2018) 4:499–504. 10.1016/j.artd.2018.09.002 30569010 PMC6288045

[13] Kurichi JE Vogel WB Kwong PL Xie D Bates BE Stineman MG. Factors associated with total inpatient costs and length of stay during surgical hospitalization among veterans who underwent lower extremity amputation. Am J Phys Med Rehabil. (2013) 92:203–14. 10.1097/PHM.0b013e31827446eb 23117271 PMC3601547

[14] Hernández C Díaz-Heredia J Berraquero ML Crespo P Loza E Ruiz Ibán MÁ. Pre-operative predictive factors of post-operative pain in patients with hip or knee arthroplasty: a systematic review. Reumatol Clínica (Engl Ed). (2015) 11:361–80. 10.1016/j.reumae.2014.12.011 25840826

[15] Lewis GN Rice DA McNair PJ Kluger M. Predictors of persistent pain after total knee arthroplasty: a systematic review and meta-analysis. Br J Anaesth. (2015) 114:551–61. 10.1093/bja/aeu441 25542191

[16] Peters ML Sommer M De Rijke JM Kessels F Heineman E Patijn J Somatic and psychologic predictors of long-term unfavorable outcome after surgical intervention. Ann Surg. (2007) 245:487–94. 10.1097/01.sla.0000245495.79781.65 17435557 PMC1877005

[17] Ye Y Pan B Gu M Fluctuation of fasting blood glucose in patients who underwent primary or revision total joint arthroplasty: a retrospective review. J Orthop Surg Res. (2020) 15:508. 10.1186/s13018-020-02029-2 33153464 PMC7643256

[18] Ko SH Bandyk DF. Interpretation and significance of ankle-brachial systolic pressure index. Semin Vasc Surg. (2013) 26:86–94. 10.1053/j.semvascsurg.2014.01.002 24636605

[19] Li YS Chen CY Lin KC Tarng YW Hsu CJ Chang WN Open reduction and internal fixation of ankle fracture using wide-awake local anaesthesia no tourniquet technique. Injury. (2019) 50:990–4. 10.1016/j.injury.2019.03.011 30904247

